# A comprehensive bioinformatics analysis to identify potential prognostic biomarkers among CC and CXC chemokines in breast cancer

**DOI:** 10.1038/s41598-022-14610-2

**Published:** 2022-06-20

**Authors:** Hossein Hozhabri, Marziyeh Mazaheri Moghaddam, Madiheh Mazaheri Moghaddam, Ali Mohammadian

**Affiliations:** 1 Institute of Biochemistry and Biophysics, University of Tehran, Tehran, Iran; 2 Department of Medical Genetics, Faculty of Medicine, Tabriz University of Medical Sciences, Tabriz, Iran; 3 Department of Genetics and Molecular Medicine, School of Medicine, Zanjan University of Medical Sciences, Zanjan, Iran; 4 Department of Medical Biotechnology, Faculty of Medical Sciences, Tarbiat Modares University, Tehran, Iran

**Keywords:** Breast cancer, Systems biology

## Abstract

Breast cancer (BC) is a major human health problem due to its increasing incidence and mortality rate. CC and CXC chemokines are associated with tumorigenesis and the progression of many cancers. Since the prognostic values of CC and CXC families' expression in various types of cancers are becoming increasingly evident, we aimed to conduct a comprehensive bioinformatics analysis elucidating the prognostic values of the CC and CXC families in BC. Therefore, TCGA, UALCAN, Kaplan–Meier plotter, bc-GenExMiner, cBioPortal, STRING, Enrichr, and TIMER were utilized for analysis. We found that high levels of CCL4/5/14/19/21/22 were associated with better OS and RFS, while elevated expression of CCL24 was correlated with shorter OS in BC patients. Also, high levels of CXCL9/13 indicated longer OS, and enhanced expression of CXCL12/14 was linked with better OS and RFS in BC patients. Meanwhile, increased transcription levels of CXCL8 were associated with worse OS and RFS in BC patients. In addition, our results showed that CCL5, CCL8, CCL14, CCL20, CCL27, CXCL4, and CXCL14 were notably correlated with the clinical outcomes of BC patients. Our findings provide a new point of view that may help the clinical application of CC and CXC chemokines as prognostic biomarkers in BC.

## Introduction

According to the GLOBOCAN 2020 data, breast cancer (BC) has the highest incidence among cancers, with almost 2.3 million new cases every year. BC is the most leading cause of cancer-related mortality, with ~ 685,000 deaths, among women worldwide^1^. There are five molecular subtypes of BC (Luminal A, Luminal B, Triple-negative/Basal-like, human epidermal growth factor 2 (HER2), and Normal-like) defined by the gene expression profile^2^. Despite advances in the therapeutic and diagnostic approaches to BC, the prognosis remains poor in some patients due to resistance to chemotherapy and metastasis. Therefore, identifying better therapeutic and prognostic biomarkers seems mandatory for BC^3,4^.

The immune mechanisms modulating cancer progression are an attractive field of interest that has taken the focus of intensive research over the years^5^. Chemokines, a family of cytokines, are secreted by tumor cells, leukocytes, immune cells, and other cell types and have been identified to regulate inflammation and immune responses^6^. Chemokines are divided into four main subgroups (CXC, CC, C, and CX3C) according to the number and location of the first two conserved cysteine residues at the N terminus^7^. Based on their functions and expression patterns, chemokines are classified into homeostatic and inflammatory subsets. Inflammatory chemokines are usually induced during inflammation. They are expressed by leukocytes and other cell types, allowing the recruitment of inflammatory leukocytes to the damaged tissues^8,9^. On the contrary, homeostatic chemokines are continuously expressed in specific tissues in the absence of apparent activating stimuli and regulate cellular trafficking and immune surveillance systems^8–10^. CC and CXC chemokines display pivotal roles in tumor angiogenesis, growth, invasion, and metastasis^7,11^. CXC chemokines can be classified into ELR^+^ (Glu-Leu-Arg) or ELR^-^ chemokines based on the presence of the tripeptide motif (Glu-Leu- Arg) at the N terminal. Of note, ELR^+^ CXC chemokines promote tumor angiogenesis, whereas ELR^-^ CXC chemokines are considered to attract lymphocytes and inhibit angiogenesis^11,12^.

Emerging studies have investigated the expression patterns and prognostic values of the CXC and CC chemokine members in a variety of human cancers, including colon cancer, gastric cancer, hepatocellular carcinoma, and non-small-cell lung cancer^13–18^. Therefore, in the current study, we aimed to conduct a comprehensive bioinformatic analysis elucidating the prognostic values of the whole CXC and CC families in BC.

## Methods

All the methods were performed in accordance with relevant guidelines and regulations.

### TCGA

The CC and CXC family expression profiles and corresponding clinical-pathological data (1104 patients) of BC patients were retrieved from The Cancer Genome Atlas (TCGA) public database (https://cancergenome.nih.gov/). The association between CC and CXC chemokines expression and clinical-pathological features and overall survival (OS) of BC patients were evaluated by the univariate and multivariate Cox regression analyses. M, N and T pathologies, pathologic_stage, progesterone receptor status, estrogen receptor status, and age at diagnosis were used in multivariate Cox analysis. All factors were considered as categorical. Age was dichomatized with respect to 55 years.

### UALCAN

UALCAN (http://ualcan.path.uab.edu/) database provides analysis of cancer-related omics data of TCGA and MET500 databases^19^. mRNA expression of CC and CXC chemokines in BC and normal tissues were analyzed using the "TCGA gene analysis" module of UALCAN database. Statistical analyses were determined by the student's t-test between groups. *P*-values of < 0.05 were considered to be statistically significant.

### Kaplan–Meier plotter

We utilized the Kaplan–Meier plotter (www.kmplot.com), an online database containing gene expression profiles and survival data of cancer patients^20^, to evaluate the prognostic value of the CC and CXC family members in breast cancer patients. In order to analyze the OS, and recurrence-free survival (RFS), samples were divided into high and low expression groups according to the median gene expression. The hazard ratios (HR) with the corresponding 95% confidence interval (CI) and log-rank *p*-value were calculated. A value of *P* < 0.05 was defined as significant.

### bc-GenExMiner v4.7

The Breast Cancer Gene-Expression Miner v4.7 (bcGenExMiner v4.7) (http://bcgenex.ico.unicancer.fr) is an online web server including expression, prognosis, and correlation modules^21^. In the current study, the "expression" module of the bcGenExMiner was applied to assess the expression of CXC and CC chemokines according to Scarff-Bloom-Richardson (SBR) grade and intrinsic molecular subtypes determined by Prediction Analysis of Microarray 50 (PAM50) test. The Dunnett–Tukey–Kramer's test and Welch's t-test were performed to calculate the *p*-value. A value of *P* < 0.05 was considered to be significant.

### cBioPortal

cBioPortal (http://www.cbioportal.org/) is a comprehensive online database for visualizing and analyzing multidimensional cancer genomics data^22,23^. The breast invasive carcinoma (TCGA, Firehose legacy) dataset, containing data from 1108 samples, was selected to analyze the genomic profile changes, including mutations, putative copy number alterations (CNAs) from genomic identification of significant targets in cancer (GISTIC), and mRNA expression Z scores (microarray). Also, the top 50 most frequently altered genes with CC and CXC chemokines in BC were obtained from cBioPortal. The *P*-value of < 0.05 was considered as the cut-off.

### STRING

The protein–protein interaction (PPI) network among two chemokine families and the top 50 frequently altered genes were constructed by STRING (https://string-db.org/) database and visualized using the Cytoscape (version 3.8.2) with a confidence score of > 0.4^24,25^.

### Enrichr

Enrichr (https://maayanlab.cloud/Enrichr/) is a web-based tool for enrichment analysis^26–28^. Enrichr was applied to perform gene ontology (GO) functional annotation (GO terms such as Biological Process and Molecular Function) and Kyoto Encyclopedia of Genes and Genomes (KEGG) pathway enrichment analysis, transcription factor analysis using Chip Enrichment Analysis (ChEA) database, and miRNA prediction using miRTarBase of CC and CXC chemokines. The ggplot2 R package was used to generate figures of KEGG analysis. *P*-value < 0.05 was set as criteria.

### MethSurv

MethSurv (https://biit.cs.ut.ee/methsurv/) is a web tool to perform survival analysis based on CpG methylation patterns^29^. MethSurv was used to identify the prognostic value of single CpG methylation of CC and CXC chemokines in BC patients.

### TIMER

Tumor IMmune Estimation Resource (TIMER) (https://cistrome.shinyapps.io/timer/) is a comprehensive resource that provides a systematic analysis of the infiltration of different immune cells and their clinical influence across a spectrum of cancer types^30,31^. In the current study, the "gene" module of TIMER was applied to examine the correlation of CC and CXC chemokines expression and immune infiltrating cells including B cell, CD8+ T cell, CD4+ T cell, macrophage, neutrophil, and dendritic cells by purity-corrected partial Spearman method. The "survival" module was also used to investigate the cumulative OS of CC and CXC chemokine expression and immune cell infiltration associated with clinical outcomes among BC patients. *P*-values < 0.05 were considered statistically significant.

#### Expression of chemokines in BC cell lines

CCLE is a database of gene expression in different cancer cell lines^32^. We assessed the expression of CC and CXC chemokines in 51 BC cell lines. We also used the data regarding age, pathology, race, and doubling times of the cell lines available in the dataset.

#### Searching for inhibitors and drug repurposing

First, the BRCA TCGA dataset was used to obtain differentially expressed genes. To search for direct competitive inhibitors of the upregulated CXCL and CCL genes, they were searched in PharmGKB^33^. This resource is a publicly available, online knowledge base integrating the effect of human genetic variation on drug response, however, it can be also used to obtain drugs targeting a gene. To search for drugs capable of reversing the direction of CC and CXC chemokines, we used the L1000Cds^2^ server. The L1000Cds^2^ perturbation database is an open resource database that provides linking gene expression patterns and drugs^34^. We searched for drugs or drug combinations that can induce opposite CC and CXC expression, to what seen in BR differentially expressed CCL and CXCL genes were used to obtain chemicals that reverse their gene expression pattern in BRCA.

## Results

### The univariate and multivariate OS analyses of CC and CXC chemokines and clinicopathological data in patients with BC

We performed univariate and multivariate Cox regression analyses to elucidate the relationships between the clinicopathological data and the survival of BC patients in TCGA. According to the multivariate analysis, age, stage, and M pathology were identified to be independent prognostic factors for BC patients (*P* < 0.05). Additionally, the univariate and multivariate Cox regression analyses clarified the prognostic significance of whole CC and CXC chemokine families in BC. The results revealed that CCL15/19/27 and CXCL7/14 expression can be independent prognostic factors for BC patients (*P* < 0.05) (Table 1).

**Table 1:** The univariate and multivariate survival analysis of the CXC and CC chemokine family and clinical-pathological data from TCGA.

Items	Univariate Cox			Multivariate Cox		
	Hazard ratio	95% CI	*P* value	Hazard ratio	95% CI	*P* value
Age	1.98	1.396–2.808	** < 0.0001**	1.035	1.020–1.051	** < 0.0001**
Stage	2.699	1.901–3.831	** < 0.0001**	2.65	1.264–5.554	**0.009**
T	1.865	1.261–2.759	**0.001**	1.032	0.582–1.827	0.913
N	2.45	1.651–3.635	** < 0.0001**	0.985	0.512–1.894	0.964
M	4.45	2.572–7.698	** < 0.0001**	3.015	1.479–6.144	**0.002**
CXCL1	1.627	1.179–2.244	**0.002**	1.256	0.744–2.120	0.392
CXCL2	1.761	1.246–2.490	**0.001**	1.406	0.799–2.475	0.236
CXCL3	1.655	1.179–2.323	**0.003**	0.853	0.543–1.338	0.489
CXCL4 (PF4)	0.71	0.452–1.116	0.136	–	–	–
CXCL5	1.74	1.112–2.721	**0.013**	1.01	0.528–1.931	0.975
CXCL6	1.652	1.171–2.331	**0.003**	0.856	0.539–1.357	0.508
CXCL7 (PPBP)	0.665	0.471–0.939	**0.019**	1.708	1.117–2.611	**0.013**
CXCL8	0.774	0.504–1.190	0.242	–	–	–
CXCL9	2.96	1.386–6.323	**0.003**	0.731	0.271–1.974	0.537
CXCL10	2.174	1.108–4.265	**0.02**	0.802	0.282–2.279	0.679
CXCL11	1.563	0.965–2.531	0.066	–	–	–
CXCL12	1.208	0.877–1.664	0.244	–	–	–
CXCL13	1.874	1.227–2.861	**0.003**	0.919	0.503–1.679	0.785
CXCL14	1.727	1.251–2.385	**0.0007**	0.53	0.344–0.816	**0.003**
CXCL16	1.623	1.169–2.254	**0.003**	0.755	0.491–1.161	0.201
CXCL17	0.798	0.569–1.120	0.192	–	–	–
CCL1	2.215	1.409–3.481	**0.0004**	0.649	0.377–1.119	0.12
CCL2	1.545	0.974–2.451	0.061	–	–	–
CCL3	0.744	0.525–1.056	0.097	–	–	–
CCL4	1.328	0.870–2.027	0.186	–	–	–
CCL5	1.978	1.247–3.137	**0.003**	0.984	0.492–1.971	0.965
CCL7	1.352	0.983–1.858	0.061	–	–	–
CCL8	0.729	0.529–1.005	0.052	–	–	–
CCL11	1.387	0.996–1.930	0.051	–	–	–
CCL13	2.111	1.169–3.812	**0.011**	0.658	0.295–1.466	0.306
CCL14	1.461	1.012–2.108	**0.041**	0.906	0.514–1.597	0.735
CCL15	1.484	1.048–2.101	**0.025**	0.64	0.423–0.968	**0.034**
CCL16	0.867	0.624–1.206	0.398	–	–	–
CCL17	1.653	1.200–2.277	**0.001**	1.212	0.765–1.920	0.411
CCL18	2.265	1.059–4.842	**0.03**	1.102	0.419–2.899	0.843
CCL19	2.051	1.459–2.882	** < 0.0001**	0.576	0.358–0.924	**0.022**
CCL20	1.787	1.163–2.746	**0.007**	0.851	0.500–1.448	0.553
CCL21	1.72	1.212–2.441	**0.002**	0.979	0.564–1.699	0.942
CCL22	1.827	1.185–2.817	**0.005**	1.064	0.597–1.896	0.832
CCL23	1.608	1.168–2.214	**0.003**	0.891	0.573–1.385	0.609
CCL24	1.354	0.903–2.030	0.14	–	–	–
CCL25	1.801	1.146–2.829	**0.009**	0.838	0.483–1.452	0.529
CCL26	1.507	1.043–2.177	**0.027**	0.897	0.577–1.395	0.632
CCL27	1.772	1.208–2.600	**0.002**	0.563	0.351–0.902	**0.017**
CCL28	1.572	1.133–2.182	**0.006**	0.895	0.595–1.346	0.597

Significant values are in bold.

### The mRNA expression analysis of CC and CXC chemokines in BC patients

**Figure 1:**
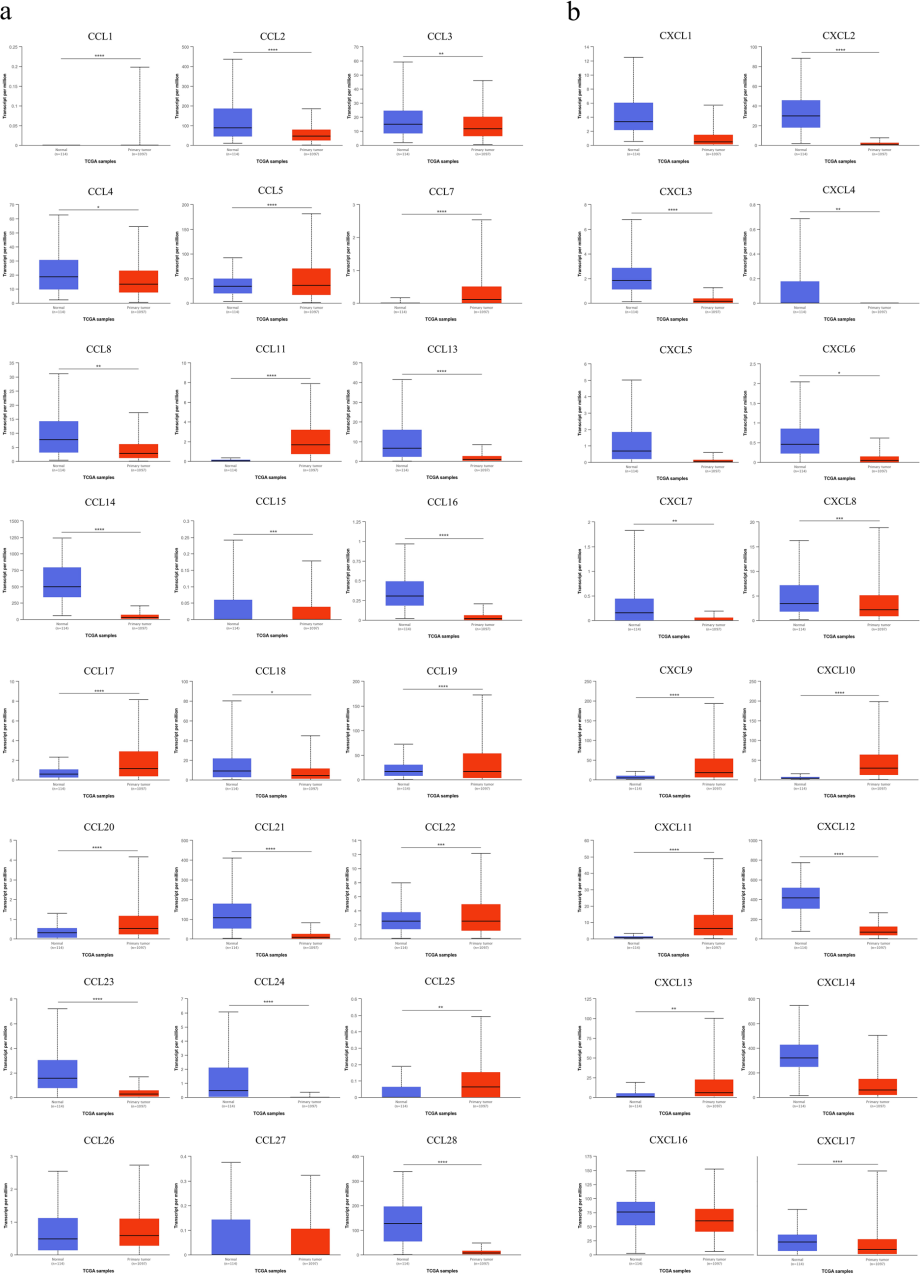
The transcription levels of CC (**a**) and CXC (**b**) chemokines in BC and normal breast tissues (UALCAN). The statistical analysis of differential expression between groups was determined by the student's t-test. The Y-axis shows transcripts per million of each RNA molecule, and the X-axis represents the samples. *P < 0.05, **P < 0.01, ***P < 0.001, ****P < 0.0001.

The mRNA expression levels of CC and CXC chemokines between primary tumor and normal tissues in BC patients were assessed using UALCAN. The mRNA expression levels of CCL1/5/7/11/17/19/20/22/25 chemokines were found to be elevated in primary tumors compared to normal specimens, while CCL2/3/4/8/13/14/15/16/18/21/23/24/28 were significantly downregulated in tumor samples (Fig. 1a). In addition, the expression of CXCL9/10/11/13 in primary tumor samples was remarkably higher than normal samples, whereas CXCL2/3/4/6/7/8/12/17 transcription levels were significantly lower (all *P* < 0.05) (Fig. 1b).

### The prognostic value of CC and CXC chemokines in patients with BC

**Figure 2:**
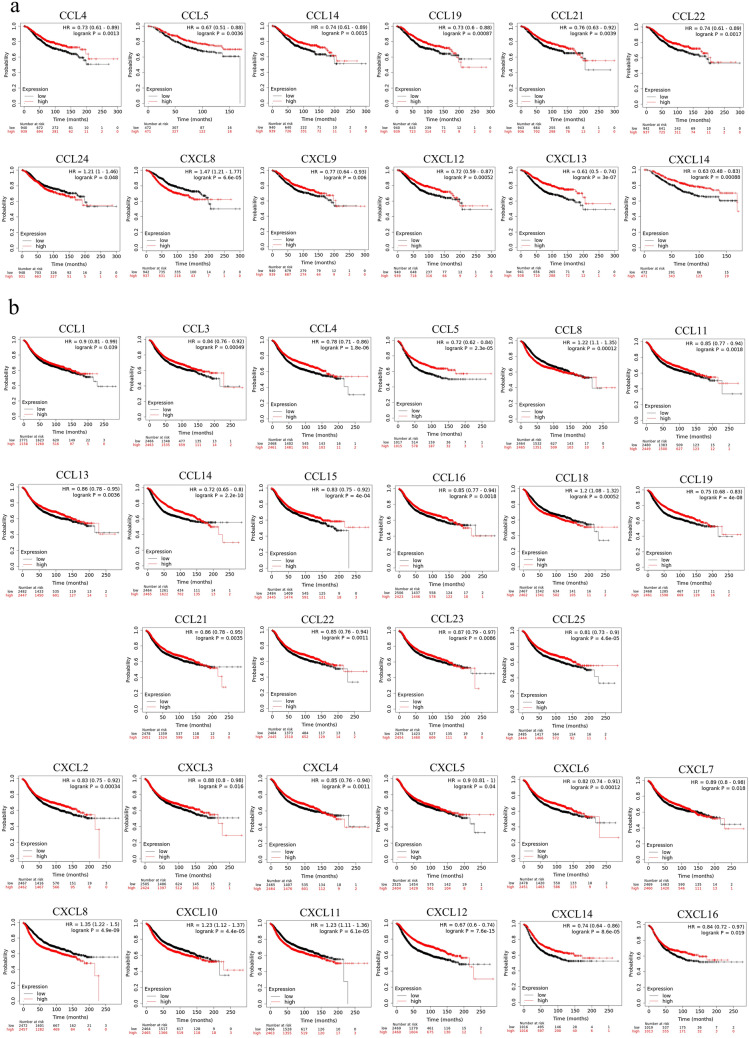
The prognostic value of the CC and CXC chemokines mRNA expression (Kaplan–Meier plotter). The association of mRNA expression of CC and CXC family with OS (**a**) and RFS (**b**) in BC patients. Red and black lines represent survival curves of the patient groups with values higher and lower than the median expression levels in the target genes, respectively. The confidence intervals are represented in brackets. HR, hazard ratio.

The Kaplan–Meier curves revealed that among CC and CXC chemokines, high mRNA expression of CCL4/5/14/19/21/22 and CXCL9/12/13/14 was notably associated with better OS (*P* < 0.05), while elevated expression of CCL24 and CXCL8 were associated with shorter OS in BC patients (*P* < 0.05). In addition, regarding RFS, BC patients with increased mRNA levels of CCL1/3/4/5/11/13/14/15/16/19/21/22/23/25 and CXCL2/3/4/5/6/7/12/14/16 were significantly correlated with favorable RFS (*P* < 0.05). On the other hand, elevated expression of CCL8/18 and CXCL8/10/11 were remarkably correlated with unfavorable RFS (*P* < 0.05). Figure 2 illustrates the Kaplan–Meier curves of chemokines in which mRNA expression levels are significantly associated with OS and RFS.

### Correlation between mRNA expression levels of CC and CXC chemokines and breast cancer grades and subtypes

**Figure 3:**
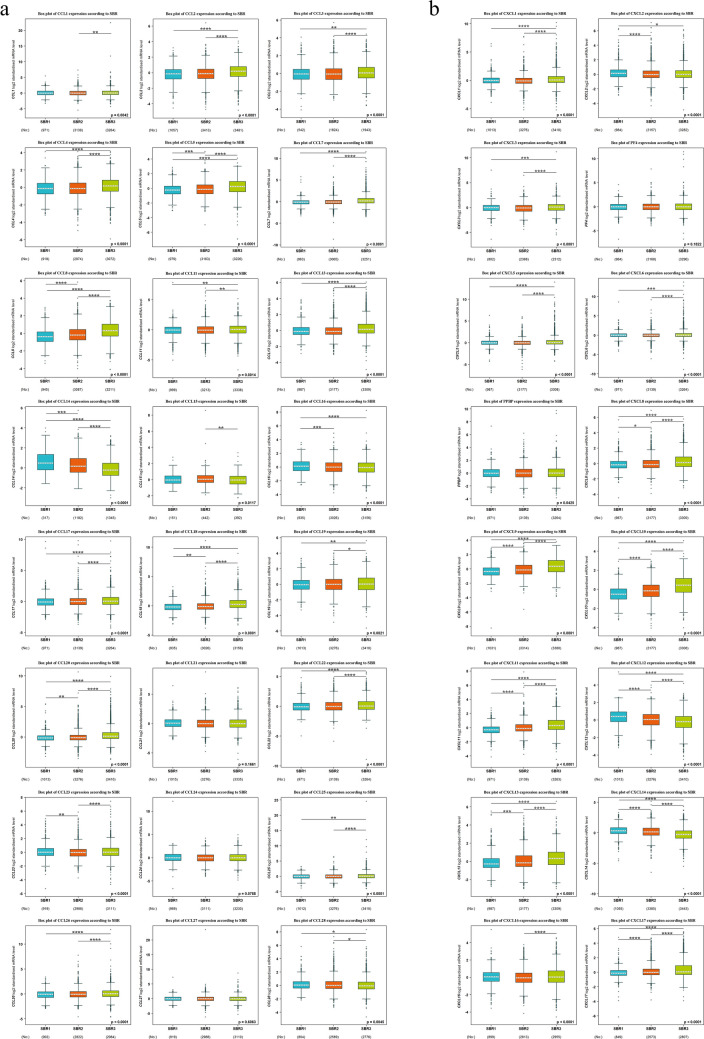
Association between CC (**a**) and CXC (**b**) chemokines expression and SBR grade status of BC patients (bc-GenExMiner v4.2). Using Welch's tests and Dunnett–Tukey–Kramer's test, the difference in mRNA expression between groups was assessed. Box plot of CC and CXC chemokines expression goes from the lower quartile (Q1) to the upper quartile (Q3), and the median is marked with a horizontal dotted line. Whiskers are lines at the bottom and the top of the box representing the distance between the quartiles and 1.5 times the interquartile range. No, number of patients. CXCL4, namely PF4. CXCL7, namely PPBP. *P < 0.05, **P < 0.01, ***P < 0.001, ****P < 0.0001.

**Figure 4:**
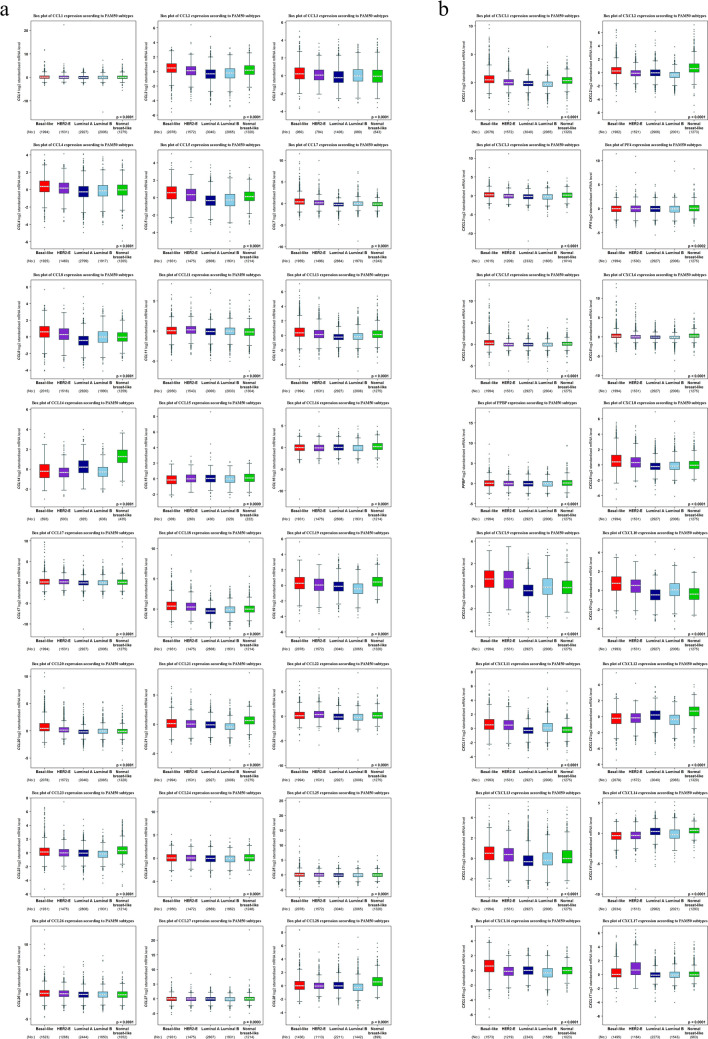
The distribution of CC (**a**) and CXC (**b**) chemokines expression according to the PAM50 subtype of breast cancer (bc-GenExMiner v4.7). Using Welch's tests and Dunnett–Tukey–Kramer's test, the difference in mRNA expression between groups was assessed. Box plot of CC and CXC chemokines expression goes from the lower quartile (Q1) to the upper quartile (Q3), and the median is marked with a horizontal dotted line. Whiskers are lines at the bottom and the top of the box representing the distance between the quartiles and 1.5 times the interquartile range. The number of patients is represented in brackets. *P < 0.05, **P < 0.01, ***P < 0.001, ****P < 0.0001.

In the current study, bcGenExMiner v4.7 was applied to compare the mRNA levels of CC, and CXC chemokines between groups of BC patients divided according to SBR grade status and PAM50 BC subtypes. Regarding the SBR grade criterion, patients with advanced SBR grades tended to express higher mRNA levels of CCL5/8/18/20 and CXCL8/9/10/11/13/17 and lower mRNA levels of CCL14 and CXCL12/14 (*P* < 0.05) (Fig. 3). In the case of PAM50 subtypes, the expression of CCL2/3/4/5/7/8/13/18/20 and CXCL1/3/5/8/10/11/13/16 was notably higher in the basal-like subtype in comparison to the HER2, luminal A/B, and normal breast-like (*P* < 0.05). Additionally, CCL11/22 and CXCL17 expression were notably highest in the HER2 subtype when compared to other subtypes (basal-like, luminal A/B, and normal breast-like (*P* < 0.05)) (Fig. 4).

### Genomic alterations and GO enrichment analysis of CC and CXC members in BC patients

**Figure 5:**
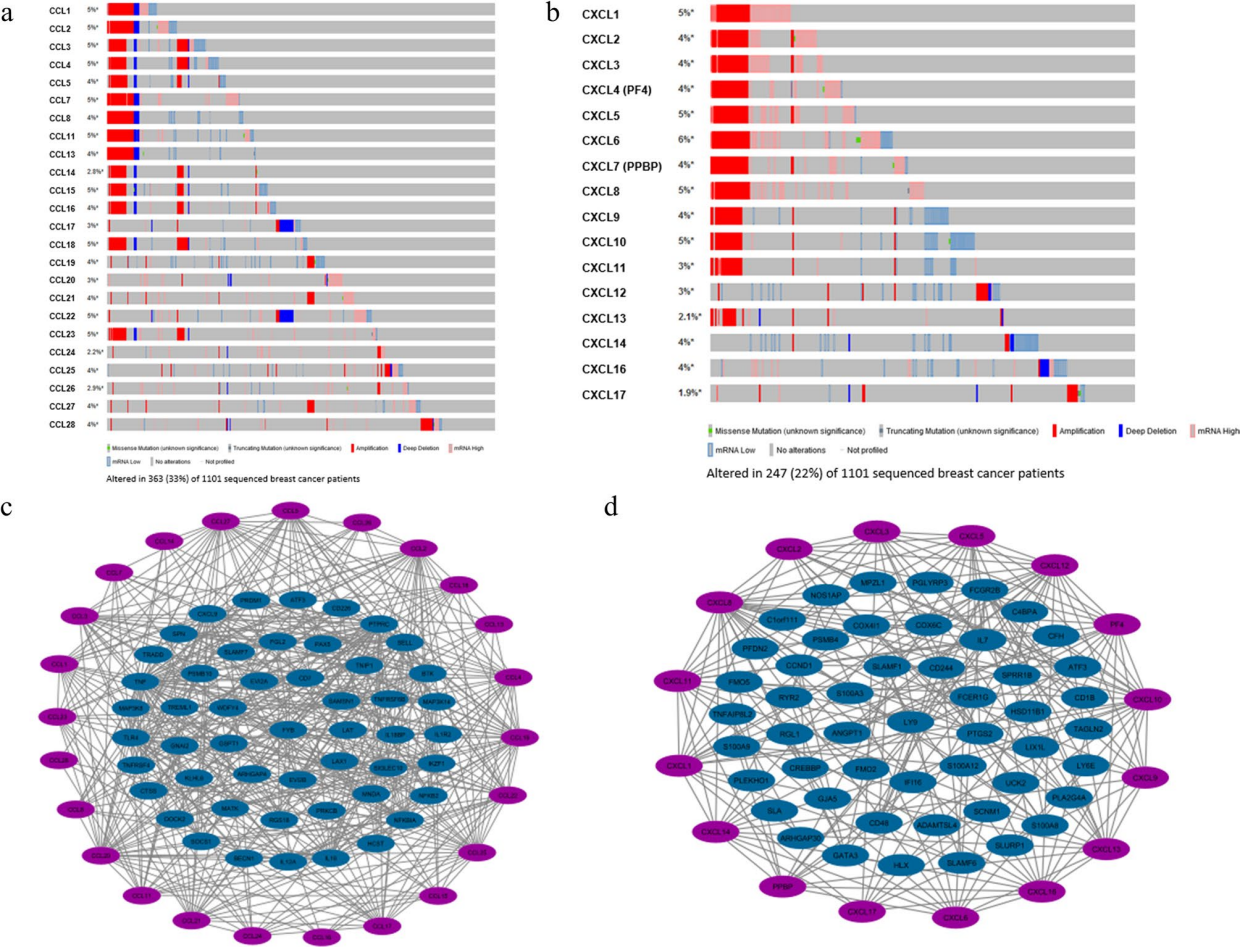
Genomic alteration of CC (**a**) and CXC (**b**) chemokines in BC patients (cBioPortal). Green in grey: missense mutation (unknown significance); darker grey in grey: truncating mutation (unknown significance); red: amplification; blue: deep deletion; pink Rectangle in grey: mRNA upregulation; blue Rectangle in grey: mRNA downregulation; grey only: no alteration. PPI network of CC (**c**) and CXC (**d**) chemokines and the 50 most frequently altered neighboring genes in BC (STRING). Gray lines represent the interactions.

Genomic alterations of the CC and CXC chemokines were analyzed using the cBioPortal database. The results showed that CC and CXC genes were altered in 363 (33%) and 247 (22%) of 1101 BC patients, respectively (Fig. 5a,b). As a result, each of CCL1/2/3/4/7/11/15/18/22/23 was altered in 5% of BC patients. Besides, our results revealed that CXCL6 was mutated in 6% of BC cases. Moreover, the 50 most frequently altered neighbor genes co-expressed with CC and CXC chemokines in BC were mapped and visualized using Cytoscape (Fig. 5c, d). Genomic alterations of the top 10 frequently altered genes with CC and CXC family members in BC patients are presented in Supplementary Tables S1 and S2.

**Figure 6:**
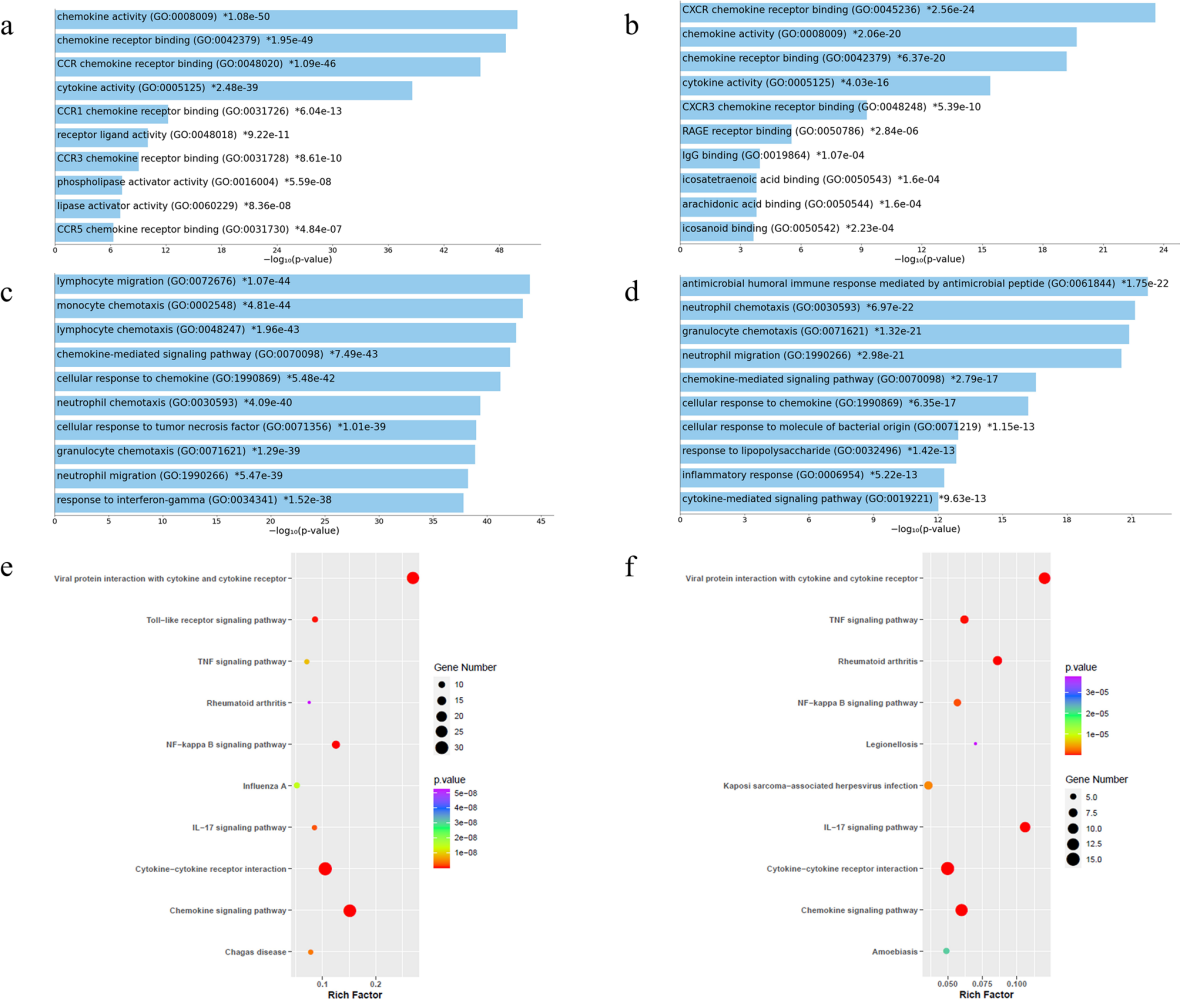
Enrichment analysis of CC and CXC chemokines and top 50 altered neighbor genes in BC. Top 10 significantly enriched GO terms of CC (**a**) and CXC (**b**) in molecular functions. Top 10 significantly enriched GO terms of CC (**c**) and CXC (**d**) in biological processes. The x-axis shows the − log10 (*P*-value), and the y-axis shows the GO terms, including molecular functions and biological processes. KEGG enrichment scatter plots of CC (**e**) and CXC (**f**) chemokines. The Y-axis shows the KEGG pathway terms, and the x-axis shows the rich factor. The *P*-value is represented by a color scale. A value of *P* < 0.05 was defined as significant.

Furthermore, the Enrichr database was used to determine the functions and pathways of CC and CXC chemokines and their frequently altered neighbor genes. The molecular functions of CC and CXC members and their neighbor genes were mainly chemokine activity and CXCR chemokine receptor binding, respectively (Fig. 6a, b). The most commonly enriched biological processes for CC and CXC members and their neighbor genes were lymphocyte migration and antimicrobial humoral immune response mediated by antimicrobial peptide, respectively (Fig. 6c, d). In KEGG pathway analysis, we revealed that CC and CXC chemokines and their neighbor genes were most commonly enriched in viral protein interaction with cytokine and cytokine receptor (Fig. 6e, f).

### Prediction of miRNA and TF associated with CC and CXC members

Transcription factors (TFs) and miRNAs potentially regulating CC and CXC chemokines were retrieved from ChEA and miRTarBase databases and summarized in Tables 2 and 3, respectively. Furthermore, the prognostic value of the resulting TFs and miRNAs were assessed using Kaplan–Meier plotter in BC. The elevated expression levels of the transcription factors IRF4, IRF8, NR1H3, PBX1, STAT6, ERG, and ESR1 were found to be significantly associated with better OS in BC patients. On the contrary, higher BP1 mRNA levels were associated with shorter OS in BC patients (all *P* < 0.05) (see Supplementary Fig. S1 online). Kaplan–Meier curves revealed that the high expression of MIR-4270, MIR-4441, and MIR-3065-5p were remarkably correlated with shorter OS, while the elevated expression of MIR-542-3p was associated with prolonged OS in BC patients (all *P* < 0.05) (see Supplementary Fig. S2 online).

**Table 2:** The most significant TFs associated with CC and CXC chemokines.

TF	Regulated gene	*P* value
**A: Upstream TFs regulating CXC chemokines**		
RELA	CXCL1, CXCL2, CXCL3, CXCL5, CXCL6, CXCL9, CXCL10, CXCL11	1.22E−06
ESR1	CXCL1, CXCL9, CXCL12	0.0049
ERG	CXCL2, CXCL3, CXCL6, CXCL10	0.0086
SUZ12	CXCL5, CXCL12, CXCL14, CXCL16	0.0159
EED	CXCL5, CXCL12, CXCL16	0.0266
**B: Upstream TFs regulating CC chemokines**		
RELA	CCL1, CCL2, CCL4, CCL5, CCL11, CCL17, CCL19, CCL20, CCL22, CCL23, CCL28	3.59E−08
PBX1	CCL3, CCL4, CCL11, CCL14, CCL15, CCL16, CCL28	0.0155
DNAJC2	CCL5, CCL7, CCL14, CCL17	0.021
BP1	CCL4	0.0213
IRF8	CCL2, CCL3, CCL4, CCL5, CCL7, CCL24	0.0275
TCF21	CCL1, CCL2, CCL8, CCL11, CCL20, CCL27	0.0275
IRF4	CCL1, CCL2, CCL11, CCL15	0.0296
CBP	CCL1, CCL2, CCL11, CCL15	0.0296
NR1H3	CCL3, CCL5, CCL20	0.0339
STAT6	CCL2	0.048

**Table 3:** The most significant miRNAs regulating CC and CXC chemokines.

miRNA	Regulated gene	*P* value
**A: Predicted miRNAs for CXC chemokines**		
hsa-mir-1-3p	CXCL1, CXCL2, CXCL3, CXCL8, CXCL12	5.82E−04
hsa-mir-100-3p	CXCL3, CXCL8	0.0034
hsa-mir-3065-5p	CXCL5, CXCL10	0.0045
mmu-mir-296-3p	CXCL11	0.0095
hsa-mir-146a-5p	CXCL8, CXCL12	0.0109
hsa-mir-4798-5p	CXCL6	0.0111
mmu-mir-871-5p	CXCL16	0.015
hsa-mir-23a-3p	CXCL8, CXCL12	0.0165
mmu-mir-27b-3p	CXCL12	0.0237
mmu-mir-210-3p	CXCL12	0.0245
**B: Predicted miRNAs for CC chemokines**		
hsa-mir-148b-3p	CCL11, CCL17, CCL19, CCL28	0.0012
hsa-let-7g-3p	CCL2, CCL5	0.0071
hsa-mir-24-3p	CCL2, CCL3, CCL4, CCL16	0.0177
hsa-mir-542-3p	CCL16, CCL22	0.0187
mmu-mir-33-5p	CCL13	0.0202
hsa-mir-548ao-3p	CCL16	0.0249
hsa-mir-4270	CCL11, CCL22	0.0276
hsa-mir-6754-5p	CCL11, CCL22	0.0278
hsa-mir-4441	CCL11, CCL22	0.0281
hsa-mir-4798-3p	CCL22	0.0295

### The prognostic value of single CpG methylation of CC and CXC chemokines in BC patients

**Figure 7:**
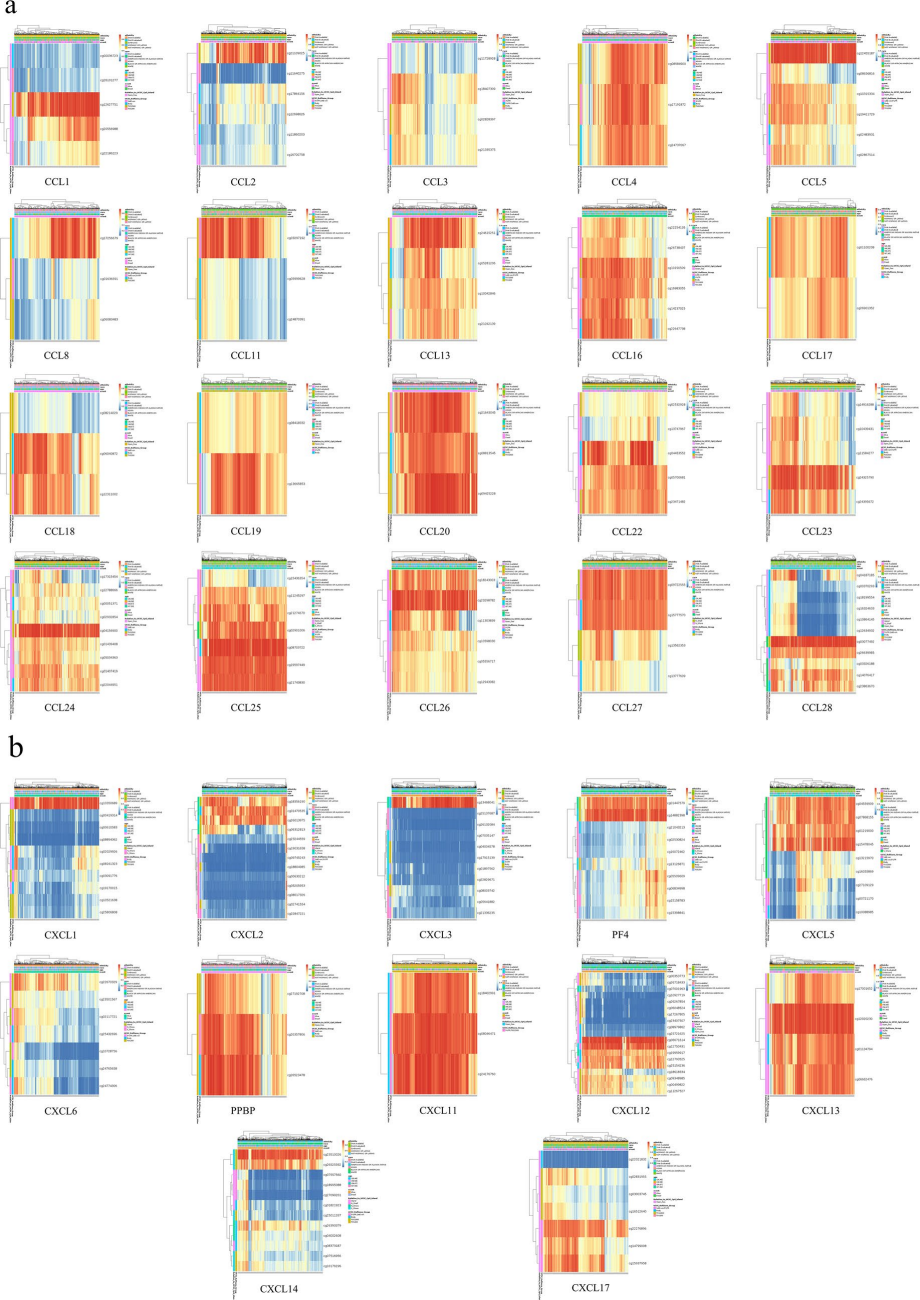
The heatmap of the CpG methylation levels of CC (**a**) and CXC (**b**) chemokines in BC patients (MethSurv). Rows indicate the CpGs, and the columns indicate the patients. Methylation levels (1 = fully methylated; 0 = fully unmethylated) are shown as a continuous variable from a red to blue color, high expression to low expression. Various colorful side boxes were used to represent the ethnicity, race, age, event, relation to UCSC_CpG_island, UCSC_refGene_Group.

The heatmaps of DNA methylation of the CC and CXC chemokines were explored (Fig. 7). Among them, cg12627751 of CCL1, cg21109025 of CCL2, cg18407309 of CCL3, cg17191872 of CCL4, cg12455187 of CCL5, cg17256679 of CCL8, cg03297192 of CCL11, cg24615251 of CCL13, cg10190509 of CCL16, cg01100208 of CCL17, cg06040872 of CCL18, cg13665853 of CCL19, cg09425228 of CCL20, cg05700681 of CCL22, cg24325790 of CCL23, cg04156900 of CCL24, cg08703722 of CCL25, cg23298782 of CCL26, cg09722555 of CCL27, cg03077492 of CCL28, cg10350689 of CXCL1, cg01470535 of CXCL2, cg13468041 of CXCL3, cg14882398 of CXCL4, cg15478045 of CXCL5, cg22670329 of CXCL6, cg20357806 of CXCL7, cg08046471 of CXCL11, cg06671614 of CXCL12, cg12020230 of CXCL13, cg23510026 of CXCL14, and cg22276896 of CXCL17 had the highest DNA methylation level. Additionally, we identified that 5 CpGs of CCL1, 2 CpGs of CCL2, 1 CpG of CCL3, 1 CpG of CCL5, 1 CpG of CCL7, 1 CpG of CCL8, 3 CpGs of CCL11, 3 CpGs of CCL13, 2 CpGs of CCL16, 2 CpGs of CCL17, 1 CpG of CCL18, 1 CpG of CCL19, 1 CpG of CCL20, 3 CpGs of CCL22, 1 CpG of CCL23, 4 CpGs of CCL24, 2 CpGs of CCL25, 1 CpG of CCL26, and 1 CpG of CCL27, 2 CpGs of CXCL1, 4 CpGs of CXCL2, 1 CpG of CXCL3, 2 CpGs of CXCL4, 5 CpGs of CXCL5, 2 CpGs of CXCL6, 1 CpG of CXCL9, 1 CpG of CXCL10, 6 CpGs of CXCL12, 2 CpGs of CXCL13, 3 CpGs of CXCL14, and 2 CpGs of CXCL17 were significantly associated with prognosis in BC patients (Table 4 and see Supplementary Figs. S3, S4 online).

**Table 4:** The significantly prognostic value of single CpG methylation of CXC and CC chemokines in BC patients (MethSurv).

Gene-CpG	HR	LR test *p*-value
CXCL1–TSS1500–N_Shore–cg08161323	0.652	0.044
CXCL1–TSS1500–N_Shore–cg19170015	0.518	0.0025
CXCL2–TSS200–Island–cg08017326	0.618	0.028
CXCL2–TSS1500–Island–cg23244559	0.638	0.024
CXCL2–1stExon; 5′UTR–Island–cg19031658	0.526	0.0038
CXCL2–TSS1500–S_Shore–cg26013975	0.521	0.0021
CXCL3–TSS200–Island–cg26132084	1.788	0.0068
PF4–Body–N_Shore–cg01447579	0.492	0.00045
PF4–1stExon; 5′UTR–Island–cg21043213	0.653	0.032
CXCL5–1stExon–Island–cg00721170	0.555	0.018
CXCL5–TSS1500–S_Shore–cg01219000	1.561	0.026
CXCL5–TSS1500–S_Shore–cg04559909	0.551	0.0062
CXCL5–TSS200–S_Shore–cg13215970	0.634	0.026
CXCL5–TSS200–S_Shore–cg16055869	0.456	0.00074
CXCL6–Body–Island–cg24765658	1.486	0.046
CXCL6–TSS200–N_Shore–cg22670329	0.515	0.0013
CXCL9–3′UTR–Open_Sea–cg03199006	1.511	0.037
CXCL12–TSS200–Island–cg00353773	1.699	0.0083
CXCL12–TSS200–Island–cg06048524	0.59	0.0089
CXCL12–TSS1500–Island–cg09348985	0.642	0.027
CXCL12–Body–N_Shore–cg19959917	0.669	0.044
CXCL12–3′UTR;Body–Open_Sea–cg12750431	1.618	0.023
CXCL12–3′; Body–Open_Sea–cg25154236	1.957	0.0058
CXCL13–3′UTR–Open_Sea–cg06662476	0.425	6.5e−05
CXCL13–TSS200–Open_Sea–cg12020230	0.63	0.036
CXCL14–5′UTR; 1stExon–Island–cg07557560	0.649	0.034
CXCL14–TSS1500–S_Shore–cg23510026	1.808	0.0098
CXCL14–TSS1500–S_Shore–cg26525592	1.703	0.0077
CXCL17–1stExon; 5′UTR–Open_Sea–cg15937958	0.59	0.0078
CXCL17–1stExon; 5′UTR–Open_Sea–cg22276896	0.635	0.026
CCL1–Body–Open_Sea–cg00036723	0.576	0.02
CCL1–TSS1500–Open_Sea–cg12627751	2.033	0.00055
CCL1–1stExon–Open_Sea–cg20556988	1.984	0.00055
CCL1–TSS200–Open_Sea–cg22186223	1.624	0.017
CCL1–TSS200–Open_Sea–cg26101277	1.666	0.01
CCL2–TSS1500–Open_Sea–cg21109025	1.865	0.0017
CCL2–5′UTR; 1stExon–Open_Sea–cg16700758	0.518	0.001
CCL3–5′UTR; 1stExon–Open_Sea–cg21335375	1.834	0.009
CCL5–1stExon; 5′UTR–Open_Sea–cg02867514	0.582	0.035
CCL7–Body–Open_Sea–cg08124722	1.958	0.0022
CCL8–TSS200–Open_Sea–cg06083483	1.887	0.0014
CCL11–TSS1500–Open_Sea–cg03297192	1.787	0.02
CCL11–TSS200–Open_Sea–cg05999628	1.628	0.015
CCL11–TSS200–Open_Sea–cg24870391	1.673	0.0096
CCL13–TSS1500–Open_Sea–cg05281206	2.25	8e−05
CCL13–TSS200–Open_Sea–cg10042846	1.576	0.031
CCL13–1stExon; 5′UTR–Open_Sea–cg24615251	1.704	0.015
CCL16–Body–Open_Sea–cg10190509	0.622	0.035
CCL16–1stExon; 5′UTR–Open_Sea–cg26738437	1.724	0.0061
CCL17–Body–Open_Sea–cg01100208	0.624	0.046
CCL17–5′UTR–Open_Sea–cg26901352	1.642	0.03
CCL18–Body–Open_Sea–cg06040872	2.013	0.00047
CCL19–3′UTR–Open_Sea–cg13665853	1.58	0.026
CCL20–TSS1500–Open_Sea–cg09425228	1.495	0.05
CCL22–Body–Open_Sea–cg04453552	0.595	0.039
CCL22–1stExon–Open_Sea–cg05700681	1.652	0.047
CCL22–TSS200–Open_Sea–cg13747967	1.564	0.026
CCL23–TSS200–Open_Sea–cg14916288	0.634	0.04
CCL24–Body–Open_Sea–cg22044951	1.728	0.0073
CCL24–1stExon–Open_Sea–cg01407419	0.642	0.033
CCL24–TSS1500–Open_Sea–cg02932854	1.625	0.024
CCL24–TSS1500–Open_Sea–cg12788666	0.611	0.025
CCL25–TSS200–S_Shelf–cg19597449	0.648	0.033
CCL25–1stExon–S_Shelf–cg21743830	0.632	0.04
CCL26–TSS1500–Open_Sea–cg12943082	1.718	0.025
CCL27–TSS200–N_Shelf–cg13562353	0.401	0.027

### Correlation between CC and CXC chemokines and immune cell infiltration in BC

We applied the TIMER database to evaluate the correlation between CC and CXC chemokines expression and immune cell infiltration in BC. The results indicated that the expression of CCL2/3/4/7/8/11/13/18/22/23/24 and CXCL9/10/11/12/16 were positively correlated with the infiltration of six immune cell types (B cell, CD8+ T cell, CD4+ T cell, macrophage cells, neutrophil cells, and dendritic cells) (all with *P* < 0.05) (see Supplementary Fig. S5 online). Moreover, we found that among CC chemokines, the expression of CCL3 was mostly correlated with high infiltration abundances of macrophage cells (Cor = 0.246, *P* = 4.82e−15) in BC. Also, CCL4 expression was mostly associated with increased infiltration abundances of neutrophils (Cor = 0.686, *P* = 3.42e−133) and dendritic cells (Cor = 0.687, *P* = 8.17e−134). We found that infiltration of CD8+ (Cor = 0.526, *P* = 1.99e−70) and CD4+ T cells (Cor = 0.597, *P* = 4.92e−94) was mainly correlated with the CCL5 expression levels. Additionally, CCL19 expression levels showed the highest correlation with B cell (Cor = 0.303, *P* = 3.76e−22) infiltration. Besides, among CXC chemokines, CXCL9 expression levels were the most highly correlated to B cell (Cor = 0.519, *P* = 1.78e−68), CD8+ T cell (Cor = 0.543, *P* = 5.79e−76), CD4+ T cell (Cor = 0.518, *P* = 4.87e−67), and dendritic cells (Cor = 0.617, *P* = 4.87e−101) infiltration in BC patients. We also found that expression levels of CXCL10 and CXCL12 had the highest association with neutrophils (Cor = 0.641, *P* = 3.08e−111) and macrophages (Cor = 0.455, *P* = 1.69e−51) infiltration, respectively. Furthermore, we utilized the Cox proportional hazard model for CC and CXC chemokines and all six tumor-infiltrating immune cells in BC. As summarized in Supplementary Tables S3 and S4, CCL5 (*P* = 0.008), CCL8 (*P* = 0.000), CCL14 (*P* = 0.042), CCL20 (*P* = 0.016), CCL27 (*P* = 0.041), CXCL4 (PF4) (*P* = 0.017), and CXCL14 (*P* = 0.004) were remarkably correlated with the clinical outcomes of BC patients.

#### The expression level of CC and CXC chemokines in BC cell lines

**Figure 8:**
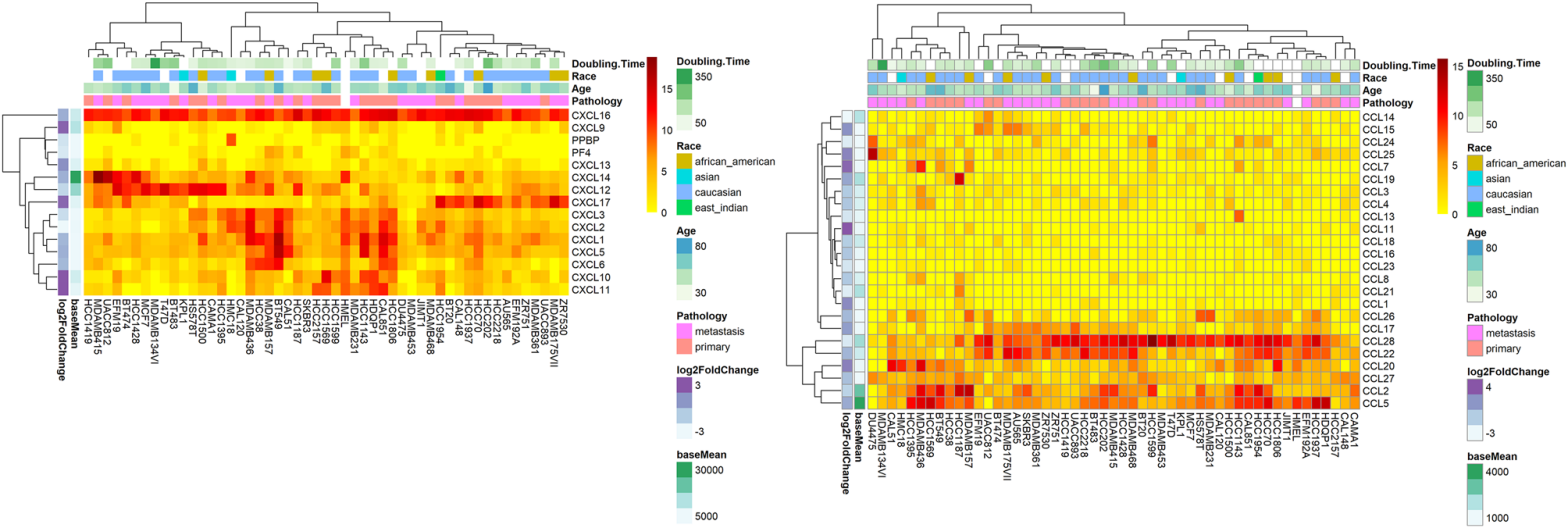
The expression patterns of CC and CXC chemokines in 51 breast cancer cell lines included in CCLE database. The information from the TCGA is also added to figure: log2 (Fold change) and mean of expression each cytokine is readable from the left annotation to each heatmap.

Heatmaps of expression values of CXC and CC chemokines in the 51 cell lines with expression information in the CCLE database are shown in Fig. 8. Race, pathology, and age of cell donors had no association with CC or CXC chemokines expression.

#### Drug candidates for reversing the effect of CC and CXC chemokines in cancer

**Figure 9:**
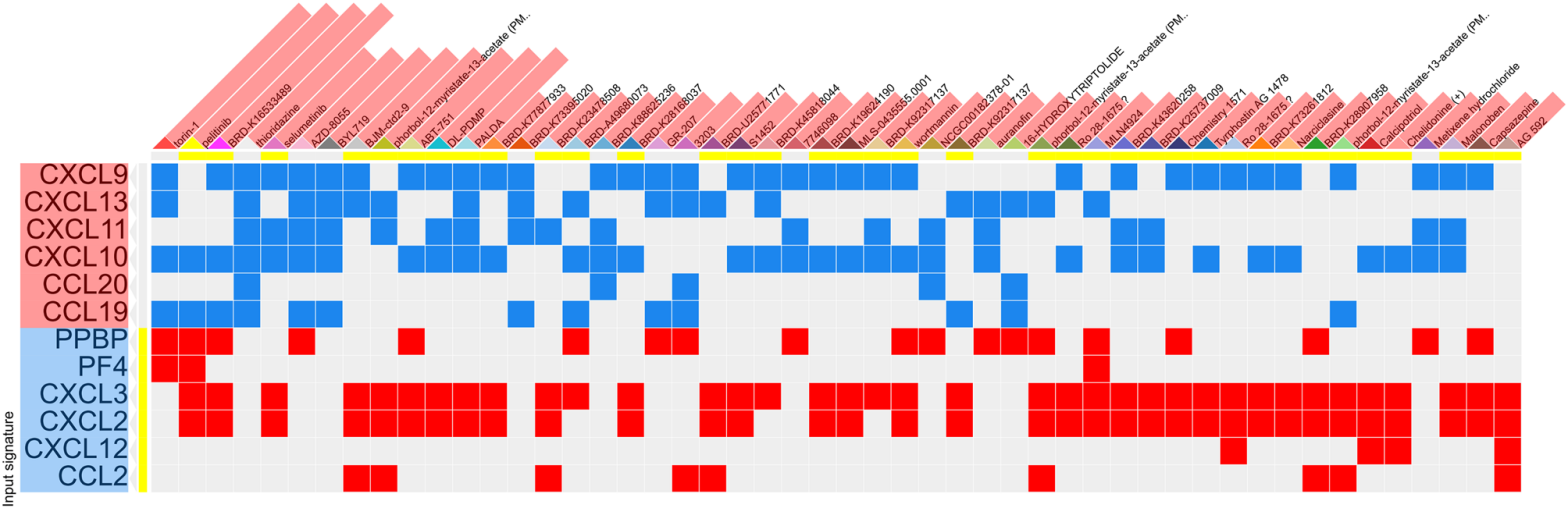
The effect of drugs on differentially expressed CC and CXC chemokines present in the L1000Cds^2^ database. Input signature is highlighted in blue and red, depending on the direction of change of the respective cytokine in TCGA breast cancer data. The abscisa lists drugs and the effect of each drug on chemokines is demonstrated by red or blue, for either up- or down-regulation of chemokines in the cells (not shown) treated by the drug (dose not shown), respectively.

**Figure 10:**
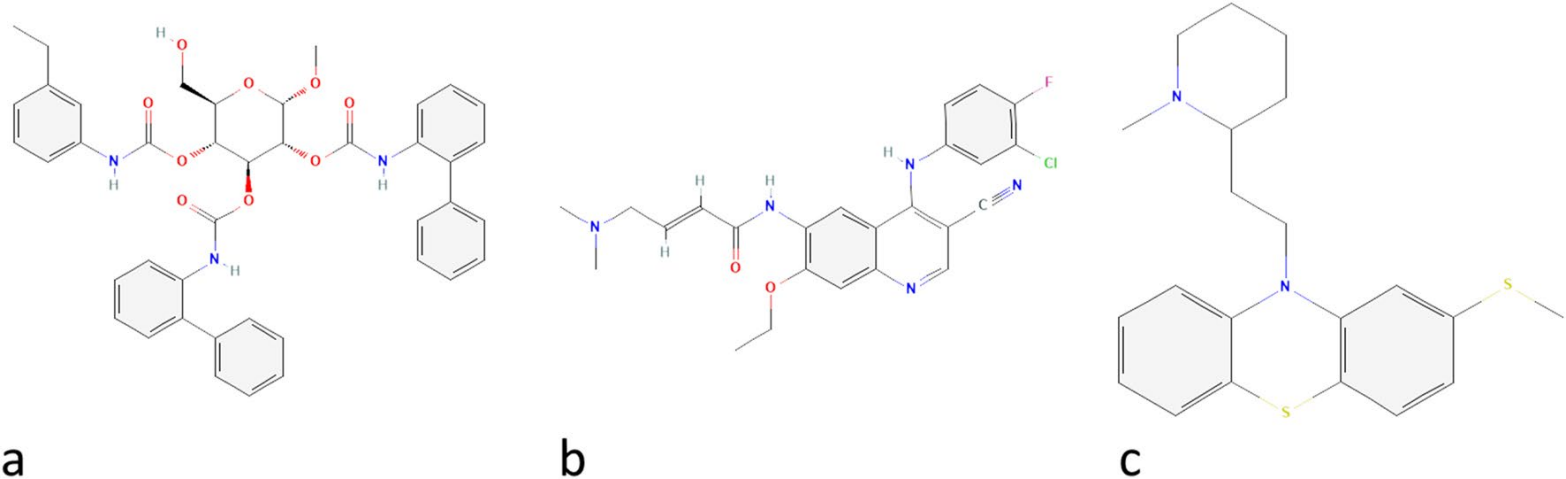
The 2D structure of top 3 drugs with signatures in the reverse of gene expression in BC patients. (**a**) BRD-K16533489, (**b**) Pelitinib, (**c**) Thioridazine.

As most drugs work through competitive inhibition, upregulated genes were submitted to https://www.pharmgkb.org/ to find current FDA approved drugs; however, there were no drugs designed to inhibit the upregulated CXCL or CCL genes. Hence, to reverse the CXCL and CCL family’s gene expression in BC, we turned to Connectivity analysis. Differentially expressed chemokines were analyzed using the L1000Cds^2^ server, and drugs and drug combinations leading to a reversal in the expression pattern of differentially regulated chemokines were obtained (Fig. 9, drug combinations not shown). The top three drugs leading to the reversal of gene expression in the opposite direction of that seen in BC are shown in Fig. 10. The top three drugs, i.e. Thioridazine, BRD-K16533489, and Pelitinib each has an overlap of 0.2857 with the reverse signature of CC and CXC chemokines in BC Furthermore, Pelitinib and Thioridazine may have a synergistic effect on restoring the gene expression pattern with an overlap of 0.4762. The 2D structure of drugs with the highest score was obtained from Pubchem^35^ and is depicted in Fig. 10.

## Discussion

Considerable data support the notion that some chemokines may promote angiogenesis and tumor progression in BC, while some may inhibit the growth and metastasis of BC cells^36^. In the current study, we applied a bioinformatic approach to elucidate the prognostic values of the whole CC and CXC chemokine families in BC. We found that the expression levels of CCL1/5/7/11/17/19/20/22/25 and CXCL9/10/11/13 in BC samples were remarkably increased compared to normal specimens. On the other hand, transcription levels of CCL2/3/4/8/13/14/15/16/18/21/23/24/28 and CXCL2/3/4/6/7/8/12/17 were significantly decreased in BC tissues.

Furthermore, we evaluated the prognostic value of the CC and CXC chemokines mRNA expression in BC patients. Our analyses revealed that enhanced expression of CCL4 is associated with favorable OS and RFS. We also found that the expression of CCL4 was higher in the basal-like subtype than the non-basal-like subtype. Unlike our findings, Sasaki et al. determined that overexpression of CCL4 in BC patients was correlated with shorter RFS. They also demonstrated that cancer cell-derived CCL4 could promote BC metastasis to the bone by bonding to the CCR5 expressed by intra-bone fibroblasts. They consequently suggested that CCL4 might display a role as a pro-metastatic mediator in the BC^37^. In the current study, we found that the high mRNA expression of CCL5 is notably associated with better OS and RFS. A growing body of evidence indicates the implication of the increased CCL5 level in BC progression and tumor metastasis promotion^38–42^. In line with our findings, Yaal-Hahoshen et al. identified the elevated expression of CCL5 in the advanced SBR grade of BC^43^. Furthermore, higher levels of CCL5 were observed in the triple-negative BC (TNBC) subtype compared to non-TNBC subtypes^41,44,45^, which is consistent with our findings. We identified that downregulation of CCL14 in BC patients is notably correlated with shorter OS and RFS, which is in line with previous findings^46^. Moreover, the CCL14 expression level was decreased with the advancement of SBR grade in BC patients. Li et al., in their study in 2011, provided clues that CCL14 might promote BC angiogenesis and metastasis^47^.

Moreover, our results showed that overexpression of CCL19 in BC patients correlated with better OS and RFS. Recently CCL19/CCR7 axis has been identified to modulate EMT and mediate tumor cell invasion and migration through the AKT signaling pathway in BC^48^. In addition, the high expression levels of CCL19 were found to predict a better prognosis^49^. Our results, like previously reported findings^50^, indicated that low expression of CCL21 is associated with a worse OS and RFS. CCL21 was identified to enhance the immunogenicity in BC^51^, while later data demonstrated that it implicates in lymphangiogenesis and metastasis^52^.

In agreement with the findings of Thomas et al.^45^, we observed that CCL22 expression level is highest in the HER2 subtype compared to other subtypes, and also the increased transcription level of CCL22 is associated with longer OS and RFS. Unlike these findings, Li et al. indicated that high expression of CCL22 is correlated with poor OS^53^. Furthermore, our analysis revealed that higher expression of CCL24 is correlated with shorter OS. Jin et al. provided clues for the contribution of the CCL24 in hepatocellular carcinoma (HCC) malignancy through the RhoB-VEGFA-VEGFR2 angiogenesis pathway. They also indicated that upregulation of CCL24 in HCC patients was associated with shorter OS^54^.

CXCL8, also known as IL-8, is a pro-inflammatory cytokine implicating in BC cell invasion and angiogenesis and promoting metastasis through the recruitment of neutrophils^41,55,56^. Higher expression of CXCL8 has been found to be associated with shorter OS in BC patients, representing a promising prognostic biomarker for OS^57–59^. Similarly, we observed an association between elevated expression of CXCL8 and shorter OS and RFS in BC patients. Consistent with our results, increased CXCL8 has been detected in the basal-like subtype^41^, and CXCL8 expression was also enhanced with the advancement of SBR grade^57^. There seem to be conflicting reports on the roles of the CXCL9 chemokine within the BC. It has been demonstrated that CXCL9-expressing tumor cells reduced tumor growth and lung metastases and prolonged survival via the recruitment of natural killer (NK) cells and T cells in murine breast cancer models^60^. Conversely, recent evidence has shown that CXCL9 promotes tumor growth and lung metastases^61^. Elevated CXCL9 level has been proved to be a good prognostic biomarker for the TNBC subgroup, while in the luminal A subgroup of patients, CXCL9 overexpression was correlated with poor prognostic characteristics^62^. Moreover, high CXCL9 expression has been related to improved survival in ER-negative BC^63^. We observed that higher CXCL9 expression appears to be associated with prolonged OS. Also, we found higher CXCL9 levels in patients with higher SBR grades. More importantly, a high expression level of CXCL9 was significantly linked to lymphocytes infiltration and better response to chemotherapy in BC patients^64–67^.

CXCL12 was proposed to enhance angiogenesis, invasion, and metastasis in BC^68–71^. However, CXCR4/CXCL12 axis was later found to be implicated in B cell-mediated killing of BC cells^72^. Moreover, Yu et al. demonstrated that enhanced CXCL12 in BC cells suppresses tumor metastasis to the lung and predicts a better prognosis^73^. Furthermore, we identified that CXCL12 expression was decreased with the advancement of SBR grade. In the case of prognosis, the results are conflicting. In an earlier study, Kang et al. indicated an inverse correlation between the expression level of CXCL12 and OS of BC patients^68^, while later data reported a positive correlation^57,74^. Similarly, we found that an elevated level of CXCL12 was correlated with prolonged OS and RFS. In contrast to these findings, Liu et al. showed that mRNA expression of CXCL12 was not associated with the OS^75^. CXCL13 is reportedly involved in BC progression and metastasis^76–78^. Xu et al. proposed that the mechanism underlying the promoting effect of CXCL13 on BC progression might be related to CXCR5/ERK pathway^79^. In line with our findings, the expression of CXCL13 was demonstrated to be enhanced in grade 2/3^80^. In the current study, the basal-like subgroup showed a dramatically higher CXCL13 level compared to other subgroups. A recent study has revealed that a high level of CXCL13 indicated longer OS and DFS in the luminal-HER2 patients^62^. Also, we identified that elevated CXCL13 conferred prolonged OS in BC patients. It has been revealed that CXCL14 inhibits tumor growth and metastasis in BC, and its protein level positively associates with OS^81^. Conversely, other data have shown that CXCL14 promotes BC tumor growth, invasion, and metastasis^82–84^. Moreover, Sjoberg et al. found that CXCL14 expression level was notably associated with reduced RFS^85^. However, Chen et al. indicated that high CXCL14 was related to better OS and favorable RFS^57^, which is in conformance with our findings. Additionally, our data in the current study identified that CXCL14 expression is negatively related to SBR grade.

Furthermore, we found that patients with advanced SBR grades tended to express higher mRNA levels of CCL8/18/20 and CXCL10/11/17. Moreover, the transcriptional levels of CCL2/3/7/8/13/18/20 and CXCL1/3/5/10/11/16 were found to be higher in the basal-like subtype in comparison to the HER2, luminal A/B, and normal breast-like subgroups. However, the mRNA levels of CCL11 and CXCL17 were obviously highest in the HER2 subtype compared to the others.

It is well documented that DNA methylation implicates in tumorigenesis^86^. DNA methylation of CC and CXC chemokines including CCL2, CCL5, CXCL2, CXCL4, CXCL5, CXCL12, CXCL14 has been identified in various cancers^87–92^. CXCL12 hypermethylation has been reported to be associated with histologically advanced disease, metastases, and a poor chance of survival in BC patients^92–95^. Also, we identified that single CpG methylation of CC and CXC chemokines was associated with prognosis in BC patients.

In the current study, we found that the high expression of MIR-4270, MIR-4441, and MIR-3065-5p was remarkably correlated with shorter OS, while the elevated expression of MIR-542-3p was associated with better OS in BC patients. A growing body of evidence has revealed that miR-542-3p plays a role as a tumor suppressor in various cancers, including ovarian and breast cancer^96–99^. Palkina et al. found that miR 3065-5p has antitumor impacts on melanoma cells^100^.

Chemokines are well known to modulate immune cell trafficking^101^. There is considerable data to support the importance of immune cell infiltration in tumor progression, which could be modulated by CC and CXC chemokines secreted by tumor and stromal cells^102^. It was previously reported that in murine breast cancer models, CXCL9-expressing tumor cells reduced tumor growth and lung metastases through the recruitment of T cells^60^. According to the data obtained from TIMER, we found that CCL3 expression levels showed the highest correlation with infiltration abundances of macrophage cells in BC among CC chemokines. Additionally, we found that CCL4 expression was mainly correlated with increased infiltration abundances of neutrophils and dendritic cells. Also, infiltration of CD8+ and CD4+ T cells was the most correlated with the CCL5 expression levels. Besides, expression levels of CCL19 had the highest association with B cell infiltration. We found that infiltration of B cell, CD8+ T cell, CD4+ T cell, and dendritic cells was mainly correlated with the CXCL9 expression levels. Additionally, infiltration abundances of neutrophils and macrophages had the highest association with CXCL10 and CXCL12 expression levels, respectively. We also found drugs that can restore the expression of the studied chemokines in BC patients. Further investigations of these drugs on BC cell lines are highly advised.

In summary, we conducted a bioinformatics analysis using public databases to evaluate the mRNA expression of the whole CC and CXC chemokines and their potential prognostic values in BC. Further in vitro and in vivo investigations are required to validate our findings. We hope our findings can provide a new point of view that may help the clinical application of CC and CXC chemokines as prognostic biomarkers in BC in the near future.

## Supplementary Information

Supplementary Information.

## Data Availability

All raw data are publicly available from corresponding databases. Processed data are available on reasonable request from corresponding author.
