# Supplementary material for: A comprehensive bioinformatics analysis to identify potential prognostic biomarkers among CC and CXC chemokines in breast cancer
Source: Sci Rep. 2022 Jun 20;12:10374. doi: 10.1038/s41598-022-14610-2 (PMC9209453; doi:10.1038/s41598-022-14610-2)
Supplement: Supplementary file 1 — Supplementary Information. [file 41598_2022_14610_MOESM1_ESM.pdf]

**Table S1.** The top 10 frequently altered genes with CC family members in breast cancer.	
**Gene symbol**	**Total alteration (%)**
CTSS	15
SLAMF7	14
LAX1	14
ATF3	13
MNDA	13
PTPRC	12
RGS18	12
SELL	11
TRADD	10
BECN1	10

**Table S2.** The top 10 frequently altered genes with CXC family members in breast cancer.	
**Gene symbol**	**Total alteration (%)**
SCNM1	23
PSMB4	20
UCK2	20
PFDN2	20
ANGPT1	20
SLURP1	19
RYR2	19
COX6C	18
GATA3	18
LY6E	18

Table S3. The cox proportional hazard model of CC chemokines and six tumor-infiltrating immune cells in BRCA.

Item	Coef	HR	95%CI_1	95%CI_u	*P*.value	Sig
B_cell	0.332	1.394	0.019	103.835	0.880	
CD8_Tcell	1.857	6.404	0.364	112.639	0.204	
CD4_Tcell	3.588	36.154	0.543	2405.776	0.094	
Macrophage	2.302	9.990	0.559	178.408	0.118	
Neutrophil	-4.572	0.010	0.000	5.814	0.157	
Dendritic	-1.305	0.271	0.026	2.851	0.277	
CCL1	-1.012	0.363	0.097	1.361	0.133	
CCL2	-0.193	0.825	0.621	1.095	0.182	
CCL3	-0.033	0.967	0.683	1.368	0.850	
CCL4	0.220	1.246	0.830	1.872	0.289	
CCL5	-0.381	0.683	0.515	0.905	0.008	**
CCL7	-0.258	0.772	0.574	1.040	0.088	
CCL8	0.734	2.082	1.507	2.878	0.000	***
CCL11	-0.059	0.942	0.750	1.184	0.610	
CCL13	-0.212	0.809	0.624	1.048	0.109	
CCL14	0.166	1.181	1.006	1.385	0.042	*
CCL15	-0.038	0.963	0.719	1.289	0.799	
CCL16	-0.256	0.774	0.366	1.635	0.502	
CCL17	0.217	1.243	0.949	1.628	0.115	
CCL18	0.084	1.088	0.943	1.256	0.250	
CCL19	-0.139	0.870	0.749	1.012	0.071	
CCL20	0.208	1.231	1.040	1.458	0.016	*
CCL21	0.025	1.026	0.909	1.158	0.681	
CCL22	-0.024	0.976	0.802	1.188	0.811	
CCL23	-0.193	0.824	0.515	1.319	0.420	
CCL24	0.391	1.479	0.979	2.234	0.063	
CCL25	-0.155	0.856	0.375	1.953	0.712	
CCL26	-0.147	0.863	0.619	1.204	0.387	
CCL27	-1.236	0.290	0.089	0.949	0.041	*
CCL28	-0.096	0.908	0.824	1.001	0.053	

*P <0.05, **P <0.01, ***P <0.001.

**Table S4.** The cox proportional hazard model of CXC chemokines and six tumor-infiltrating immune cells in BRCA.						
**Item**	**Coef**	**HR**	**95%CI_1**	**95%CI_u**	***P*.value**	**Sig**
B_cell	-1.972	0.139	0.002	12.667	0.392	
CD8_Tcell	0.601	1.825	0.121	27.411	0.664	
CD4_Tcell	3.143	23.162	0.417	1287.200	0.125	
Macrophage	1.701	5.478	0.335	89.703	0.233	
Neutrophil	-1.182	0.307	0.001	94.108	0.686	
Dendritic	-0.271	0.763	0.090	6.466	0.804	
CXCL1	-0.206	0.814	0.598	1.107	0.190	
CXCL2	-0.042	0.959	0.753	1.220	0.731	
CXCL3	-0.190	0.827	0.463	1.478	0.521	
PF4	0.614	1.848	1.118	3.056	0.017	*
CXCL5	0.023	1.024	0.696	1.506	0.906	
CXCL6	0.044	1.045	0.721	1.515	0.815	
PPBP	0.045	1.046	0.588	1.863	0.878	
IL8	0.067	1.069	0.930	1.229	0.347	
CXCL9	-0.169	0.844	0.706	1.009	0.063	
CXCL10	0.143	1.154	0.835	1.596	0.386	
CXCL11	0.078	1.081	0.771	1.515	0.653	
CXCL12	0.019	1.019	0.841	1.234	0.849	
CXCL13	-0.046	0.955	0.878	1.040	0.291	
CXCL14	-0.117	0.889	0.821	0.964	0.004	**
CXCL16	-0.231	0.794	0.604	1.044	0.098	
CXCL17	0.018	1.018	0.950	1.091	0.617	

**P* <0.05, ***P* <0.01, ****P* <0.001.

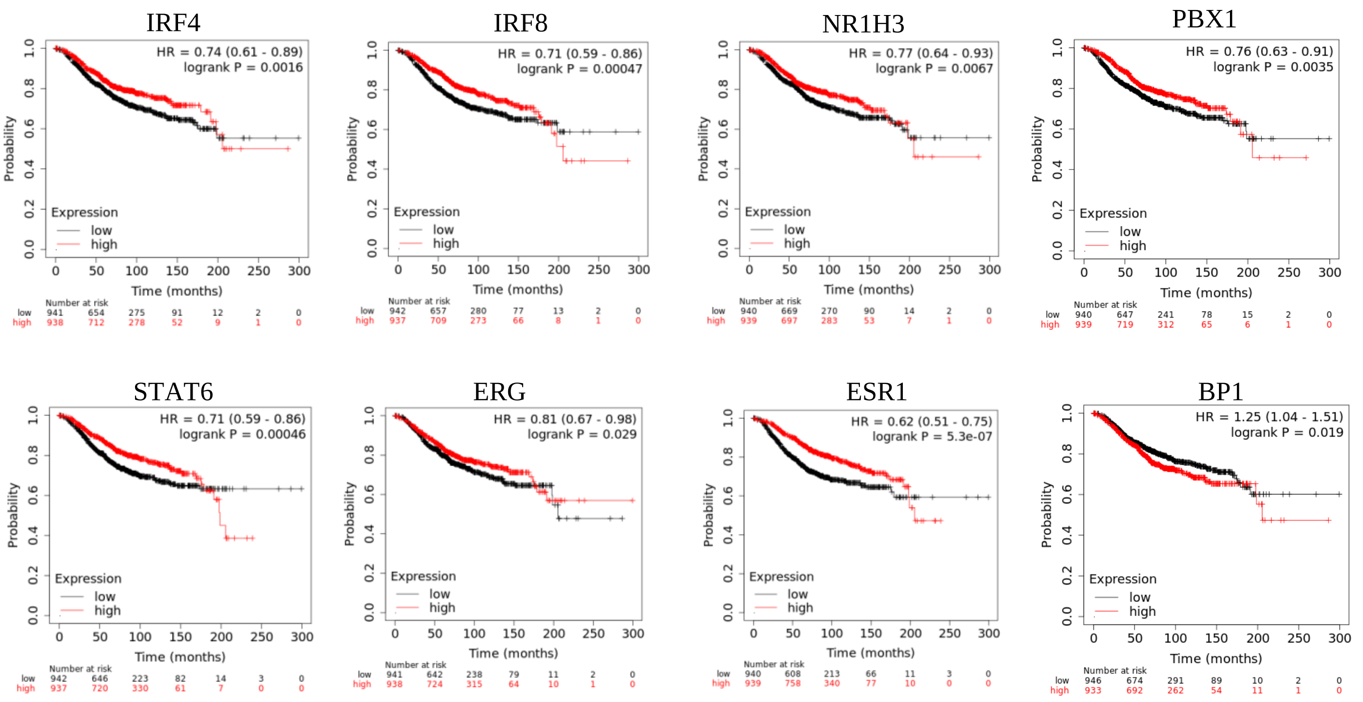

Supplementary Fig. S1. The prognostic value of the TFs regulating CC and CXC chemokines (Kaplan-Meier plotter). The association of mRNA expression of IRF4, IRF8, NR1H3, PBX1, STAT6, ERG, ESR1, and BP1 with OS in BC patients. A value of *P* < 0.05 was defined as significant. The confidence intervals are represented in brackets. Red: high expression level; black: low expression level. HR, hazard ratio.

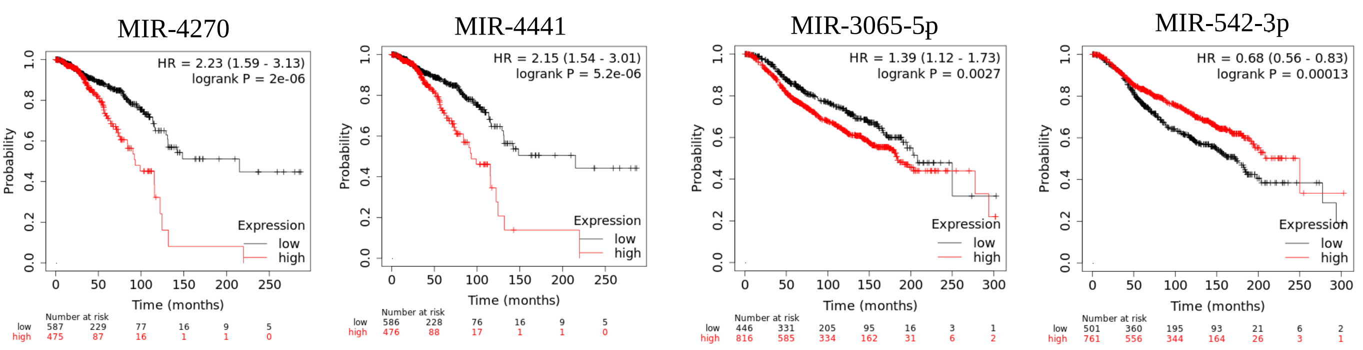

Supplementary Fig. S2. The prognostic value of the miRNAs regulating CC and CXC chemokines (Kaplan-Meier plotter). The association of MIR-4270, MIR-4441, MIR-3065-5p, and MIR-542-3p expression with OS in BC patients. A value of P < 0.05 was defined as significant. The confidence intervals are represented in brackets. Red: high expression level; black: low expression level. HR, hazard ratio.

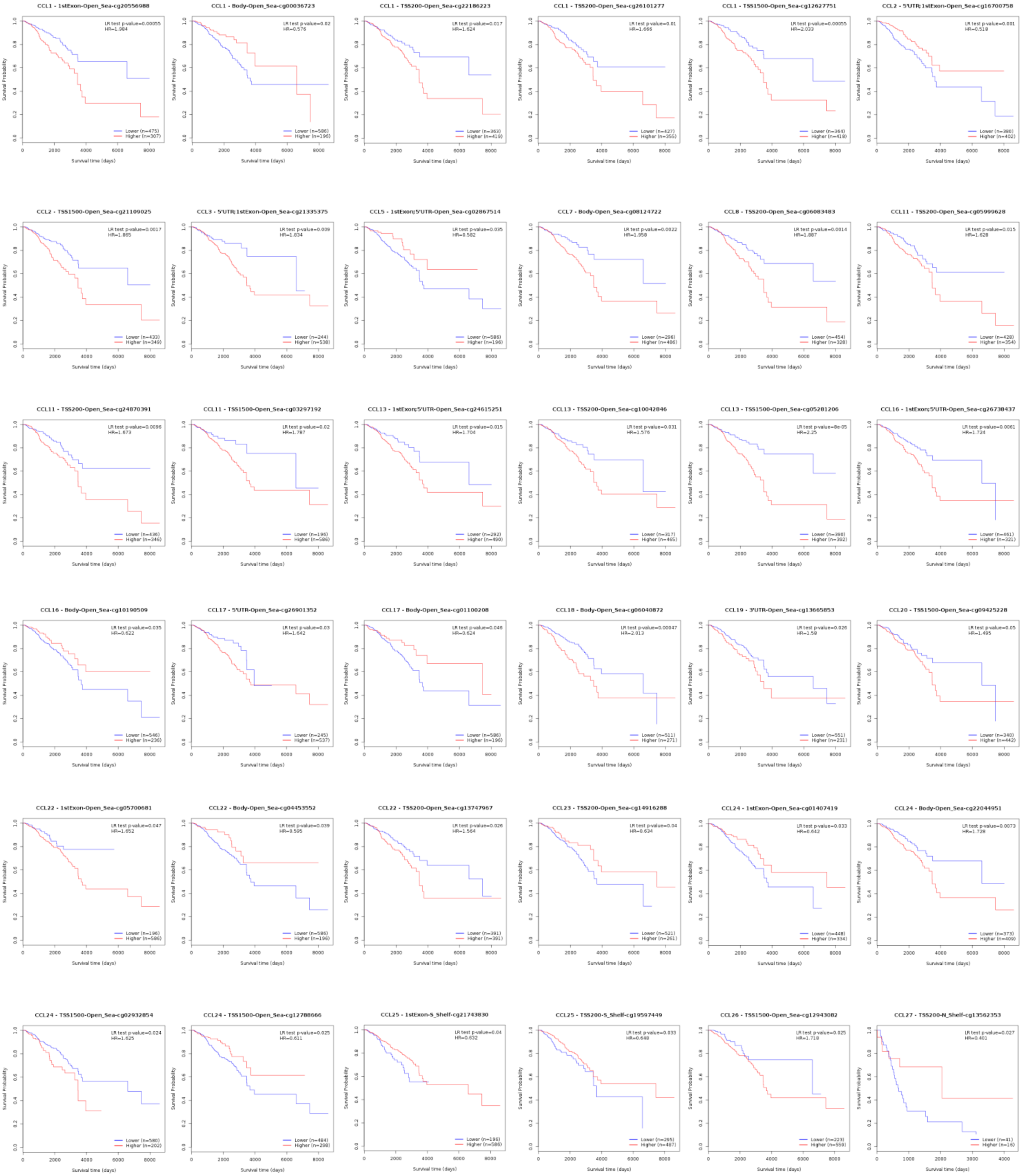

Supplementary Fig. S3. Kaplan–Meier curves for visualizing the prognostic value of single CpG methylation of CC chemokines in BC patients.

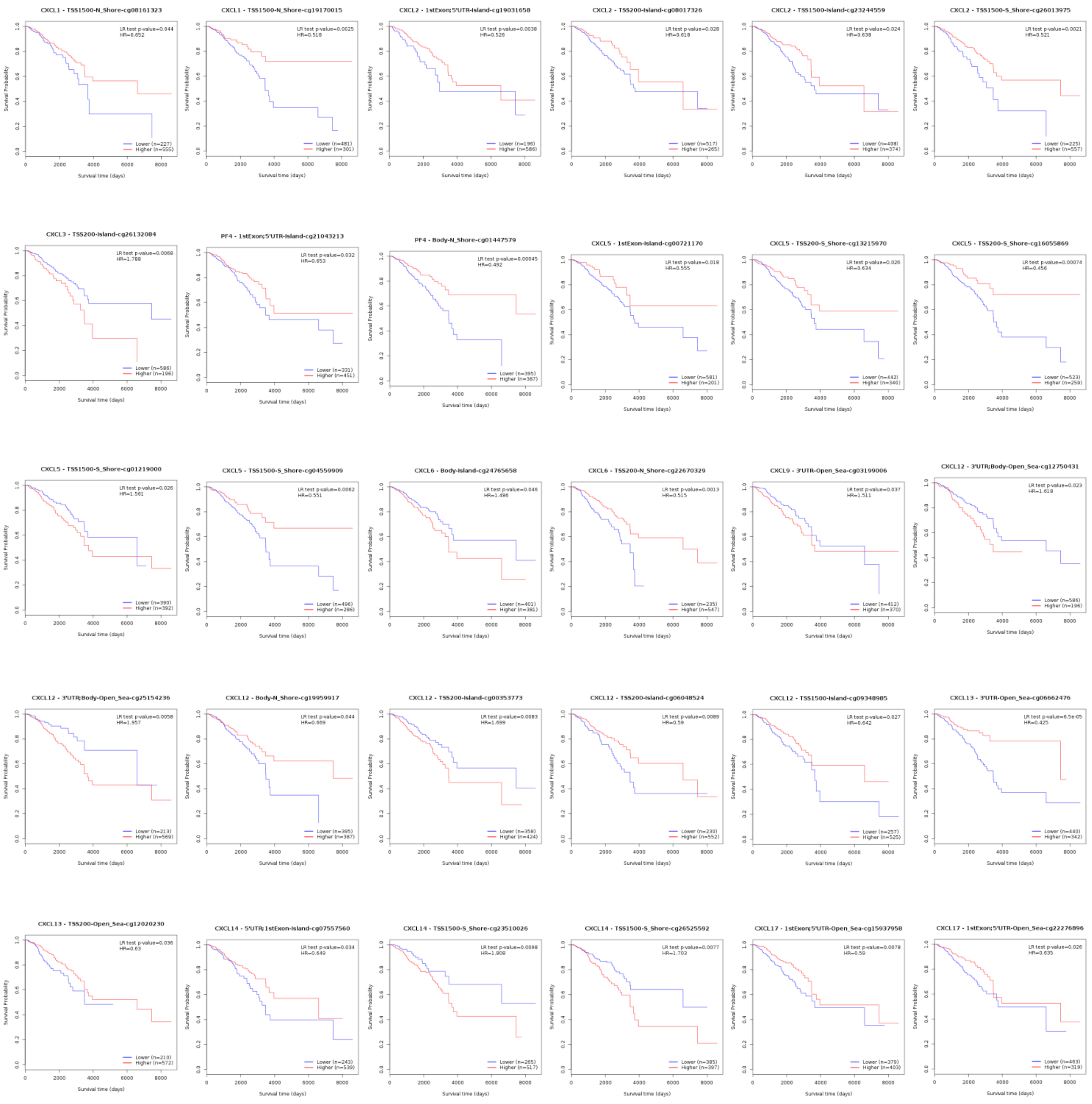

Supplementary Fig. S4. Kaplan–Meier curves for visualizing the prognostic value of single CpG methylation of CXC chemokines in BC patients.

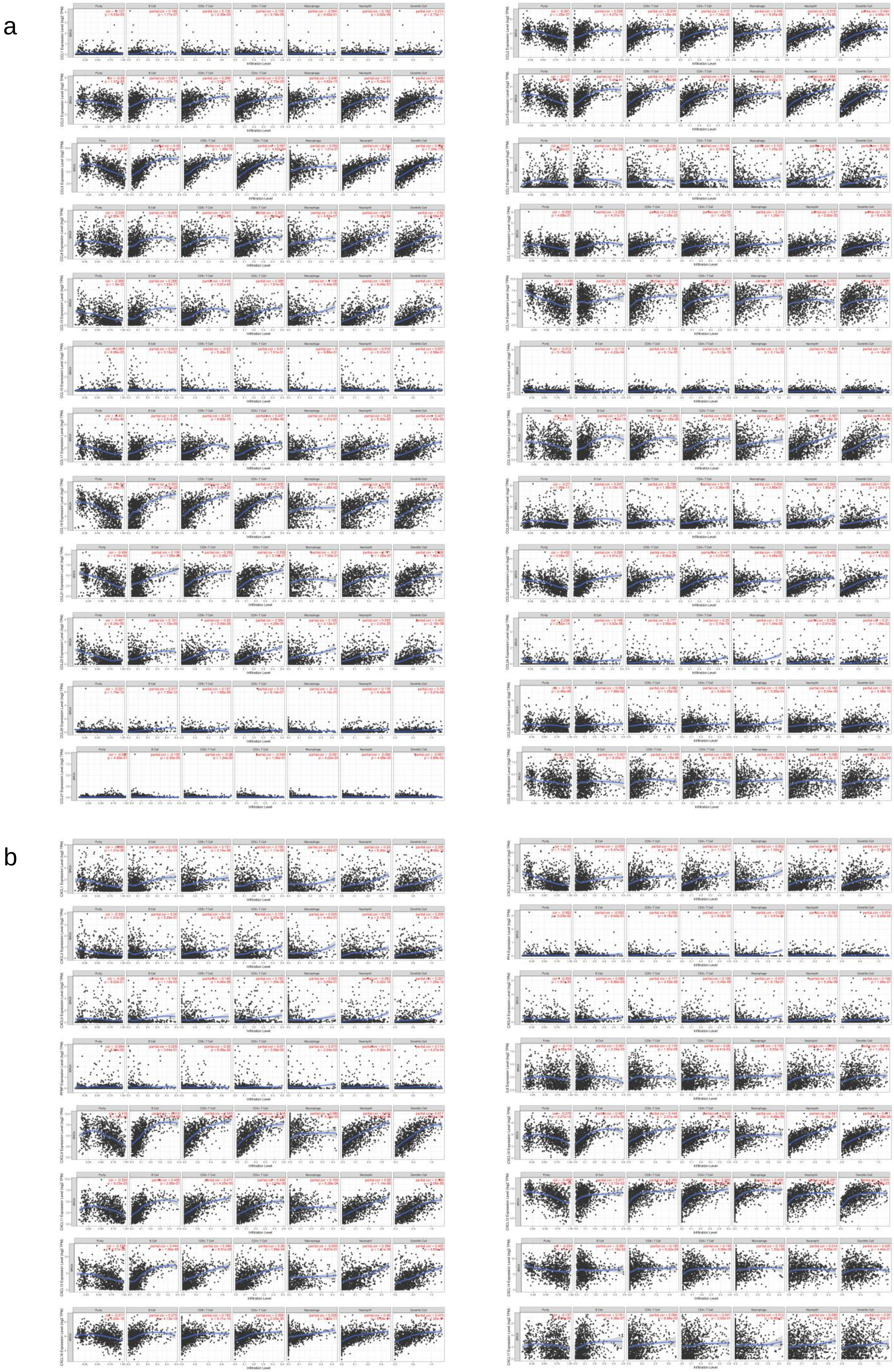
a
b

Supplementary Fig. S5. The correlation between CC (**a**) and CXC (**b**) chemokines expression and immune cell infiltration in BC (TIMER). CXCL8, namely IL8.
